# Exploring Biomineralization in Echinoderms: A Review of Gene Regulatory Networks Through Bibliometrics and Comparative Genomics

**DOI:** 10.3390/biology15201796

**Published:** 2026-10-09

**Authors:** Xueying Guo, Xiao Cong, Zhenglin Yu, Xiutang Yuan

**Affiliations:** 1 Yantai Institute of Coastal Zone Research, Chinese Academy of Sciences, Yantai 264003, China; guoxueying23@mails.ucas.ac.cn (X.G.); xiaocong@yic.ac.cn (X.C.); zlyu@yic.ac.cn (Z.Y.); 2 University of Chinese Academy of Sciences, Beijing 100049, China

**Keywords:** echinoderm, genomics, biomineralization genes, primary mesenchyme cells, gene regulatory networks

## Abstract

Biomineralization is a defining feature of echinoderms and underlies the formation of their calcitic endoskeletons. Considerable progress has been made in understanding the molecular basis of echinoderm biomineralization, particularly in sea urchins. However, studies of other echinoderm classes remain limited, and a comprehensive comparison of biomineralization genes and gene regulatory networks across the phylum is still lacking. Here, we combine bibliometric analysis with comparative genomic evidence to summarize current advances in echinoderm biomineralization research. Bibliometric analyses reveal the development of the field, major research themes, and emerging directions, whereas comparative genomic analyses provide an overview of biomineralization genes and gene regulatory networks across different echinoderm classes. Comparative evidence indicates that many transcription factors associated with skeletogenesis are widely distributed across echinoderms. In contrast, biomineralization genes exhibit substantial variation among echinoderm classes. These similarities and differences suggest that broadly shared developmental regulators have been integrated with lineage-specific biomineralization genes to generate the diversity of echinoderm skeletal structures. This review provides a comprehensive overview of biomineralization genes and gene regulatory networks in echinoderms and establishes a comparative framework for future studies of skeletal development, gene regulation, and the evolution of biomineralization across the phylum.

## Simple Summary

Echinoderms, including sea urchins, sea cucumbers, starfish, brittle stars, and crinoids, produce mineralized skeletons that vary greatly in form among different groups and developmental stages. Understanding how these skeletons form is important for revealing the biological mechanisms underlying skeletal development and diversity. This review summarizes the development and major research themes of echinoderm biomineralization studies and examines the genes involved in skeletal formation across different echinoderm groups. We show that many genes controlling skeletal development are broadly shared among echinoderms, whereas some genes directly involved in mineral formation vary considerably among groups. By integrating previous biological studies with literature-based and genomic comparisons, this review provides a broader understanding of the molecular basis of echinoderm biomineralization and highlights important questions for future research beyond the extensively studied sea urchins.

## 1. Introduction

Biomineralization refers to a biological process in which organisms form inorganic minerals under tight biological control [1]. It occurs in both prokaryotes and eukaryotes and generates a wide range of minerals, including silica, calcium carbonate, calcium phosphate, and iron oxides [2,3]. Through cellular activities and organic macromolecules, organisms regulate ion transport, mineral nucleation, crystal growth, polymorph selection, and the spatial distribution of mineral deposition [4,5,6]. The resulting structures perform important functions in mechanical support and protection [7]. In marine animals, representative biomineralized structures include mollusk shells, crustacean exoskeletons, and echinoderm skeletons [8]. Their considerable structural and chemical diversity makes marine organisms important models for studying the formation and regulation of biominerals.

**Figure 1:**
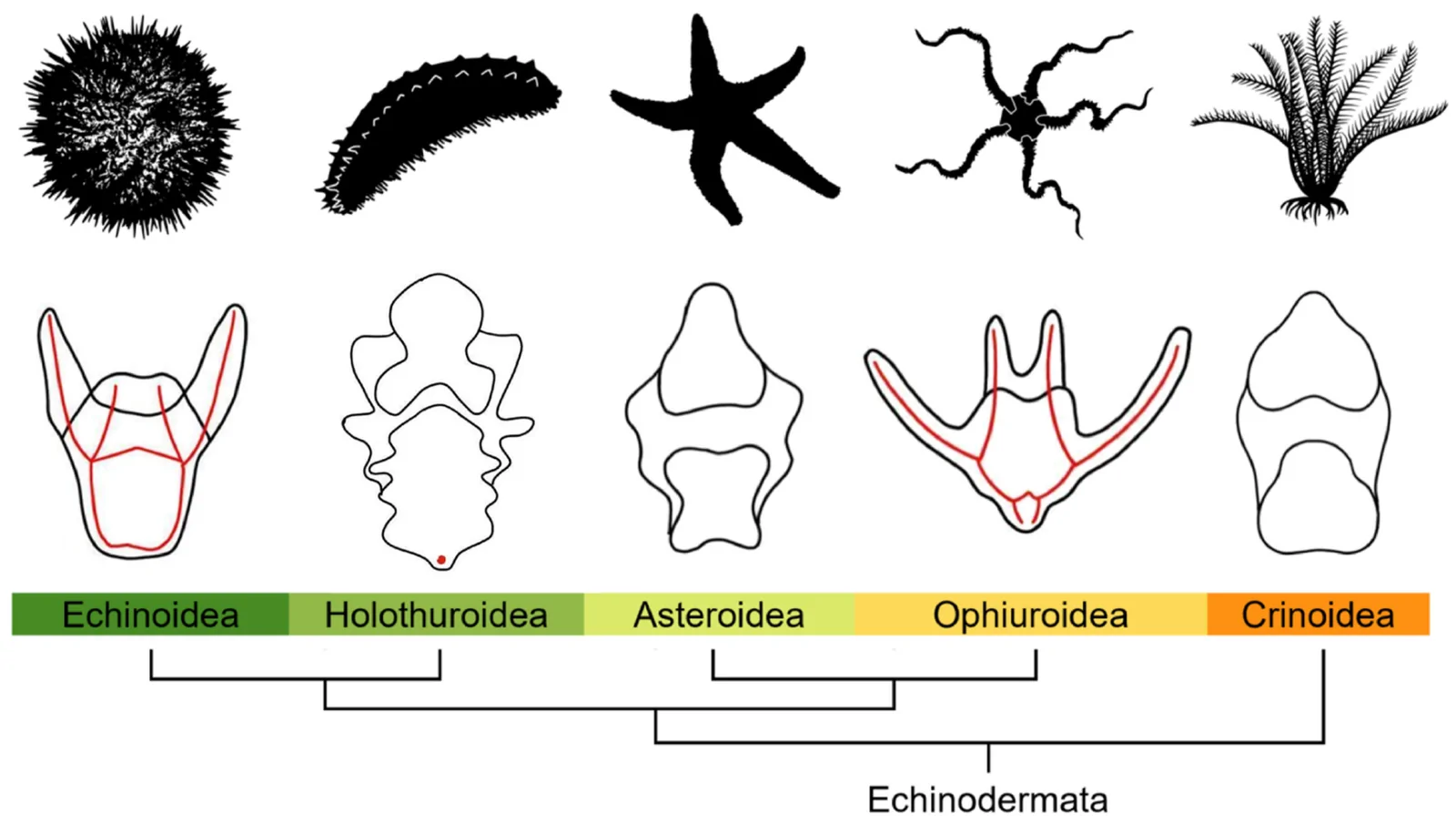
Phylogeny and larval skeletal diversity of Echinodermata. The five extant echinoderm classes are shown with representative adult silhouettes and larval schematics. The phylogenetic topology follows [18]. Pluteus larvae in Echinoidea and Ophiuroidea possess elongated arms supported by spicules. Auricularia-type larvae are found in other groups of echinoderms. Among these, holothuroid larvae possess reduced skeletal elements, whereas the asteroid and crinoid larvae shown lack mineralized skeletal elements. The holothuroid auricularia schematic was redrawn with reference to published morphological descriptions and illustrations [16]. The larval skeletons are highlighted in red. Larval schematics were created by the authors. Adult silhouettes were obtained from images available through PhyloPic (https://www.phylopic.org/): Echinoidea, Caleb M. Gordon (CC BY 4.0); Holothuroidea, Lauren Sumner-Rooney and Kurtis Wothe (CC0 1.0); Asteroidea, Hans Hillewaert and T. Michael Keesey (CC BY-SA 3.0); Ophiuroidea, Kurtis Wothe (CC0 1.0); and Crinoidea, Guillaume Dera (CC0 1.0).

Unlike the external shells or exoskeletons produced by many marine invertebrates, echinoderms form a mesoderm-derived endoskeleton beneath the epidermis, composed primarily of magnesium calcite [9,10,11]. The phylum Echinodermata comprises five extant classes: Echinoidea, Holothuroidea, Asteroidea, Ophiuroidea, and Crinoidea. Echinoderms generally undergo indirect development, in which a bilaterally organized larva undergoes metamorphosis to form a pentaradial juvenile that subsequently develops into the adult form. Larval and adult skeletal structures can differ markedly in morphology, developmental origin, and function, providing different developmental contexts for biomineralization [12,13]. Although adult endoskeletons are widespread across echinoderm classes, larval mineralized structures vary markedly among lineages [11,14]. Echinoids and ophiuroids develop prominent mineralized skeletons in their pluteus-type larvae, whereas holothuroid auricularia-type larvae generally form only small and reduced skeletal elements [15,16,17]. In contrast, asteroid larvae lack mineralized structures, and an auricularia-like developmental stage has also been reported in at least one crinoid species (Figure 1) [18,19]. Given that it is present in both Hemichordata, the sister group of echinoderm, and Crinoidea, the sister group to the remaining extant echinoderm classes, the auricularia is recognized as the ancestral larval morphotype, with the pluteus representing a derived morphotype [15,18]. Intriguingly, pluteus larvae with well-developed larval skeletons occur in echinoids and ophiuroids, which are phylogenetically separated within Echinodermata [14,15,20]. This distribution has led to two alternative evolutionary scenarios: larval skeletons may have been acquired independently in echinoids and ophiuroids, or they may have originated earlier and subsequently been reduced or lost in other lineages. Current comparative evidence does not fully resolve these alternatives [15,18]. The marked differences in skeletal morphology and larval skeletogenesis among echinoderm classes provide a useful comparative framework for investigating the conserved and variable molecular mechanisms underlying biomineralization.

Developmental gene regulatory networks (GRNs) provide a framework for explaining how genomic regulatory information establishes cell identity and ultimately controls morphogenesis [21]. Among echinoderms, this framework has been developed most extensively in sea urchins, in which the embryonic skeletogenic GRN links cell fate specification to the effector processes that produce the larval skeleton [12]. However, current knowledge is strongly concentrated in a few model euechinoids, particularly *Strongylocentrotus purpuratus* and other camarodont species, whereas molecular evidence from non-model echinoids and the other echinoderm classes remains much more limited [22,23]. Comparative studies have revealed both conserved and divergent features of skeletogenic regulation, but molecular evidence remains uneven across echinoderm groups, and experimentally validated regulatory interactions have been characterized in only a limited number of taxa [12,24,25]. Consequently, the extent to which regulatory components identified in sea urchins are conserved and developmentally deployed across echinoderm classes remains incompletely understood, limiting our ability to relate molecular variation to differences in larval skeletogenesis and skeletal morphology [12,26].

Comparative genomics provides a useful biological perspective by comparing homologous genes across species and identifying broadly distributed and lineage-associated patterns in gene repertoires [27]. Previous reviews have summarized the molecular mechanisms underlying echinoderm skeletogenesis, with particular emphasis on the skeletogenic cell lineage and gene regulatory networks in sea urchin [9,12,13,22,28,29,30,31]. Comparative studies have also identified lineage-specific variation and a conserved skeletogenic gene toolkit across several echinoderm classes [30,32]. However, existing comparisons remain incomplete in several respects: they have generally covered only a limited number of representative species, and they have focused on selected biomineralization genes or particular regulatory gene families rather than a comprehensive and consistently defined set of candidate genes. Thus, despite the increasing availability of genome assemblies and annotations from a broader range of echinoderm taxa, previous comparisons have remained limited in taxonomic coverage, species sampling, and the breadth of genes examined. In addition, echinoderm biomineralization has been investigated from diverse perspectives, making a broader assessment of the field useful for understanding its overall development and research focus. Bibliometric analysis can quantitatively characterize publication trends, collaboration networks, and changes in major research themes over time [33,34]. However, to our knowledge, no bibliometric study has specifically focused on echinoderm biomineralization. Accordingly, the present review extends previous syntheses by integrating a bibliometric overview of the field with a broader comparison of biomineralization genes across echinoderm classes.

Here, we first used bibliometrix and CiteSpace to provide a field-level overview, characterizing publication trends, collaboration patterns, influential studies, keyword structure, and changes in major research themes within the literature analyzed here [35,36]. Building on this broader overview, we further synthesized current knowledge of the cellular and molecular regulation of echinoderm skeletogenesis and surveyed biomineralization-related genes in 14 species representing all five extant echinoderm classes, focusing on their taxonomic distribution. Together, these complementary analyses aim to identify uneven research attention and major knowledge gaps, distinguish molecular components that are broadly distributed from those showing lineage-associated variation, and provide a comparative basis for future functional studies of echinoderm biomineralization.

## 2. Data Sources and Methods

### 2.1. Literature Collection

Data for bibliometric analysis were systematically retrieved from the Web of Science (WoS) Core Collection database, with the final search conducted on 7 October 2025. The WoS Core Collection indexes included in the search were Arts and Humanities Citation Index (A&HCI), Book Citation Index–Social Sciences and Humanities (BKCI-SSH), Book Citation Index–Science (BKCI-S), Current Chemical Reactions (CCR-EXPANDED), Emerging Sources Citation Index (ESCI), Index Chemicus (IC), Conference Proceedings Citation Index–Social Sciences and Humanities (CPCI-SSH), Conference Proceedings Citation Index–Science (CPCI-S), Science Citation Index Expanded (SCI-EXPANDED), and Social Sciences Citation Index (SSCI). Boolean Search Logic was applied in the “Topic” field (TS), which includes the title, abstract, author keywords, and Keywords Plus^®^. The complete search string was as follows:

TS = (echinoderm* OR starfish* OR “sea urchin*” OR “sea cucumber*” OR “brittle star*” OR crinoid*) AND TS = (biomineralization OR biomineralize* OR skeleton* OR skeletal* OR calcification OR “gene regulatory network”) AND TS = (gene* OR protein* OR cellular OR molecular OR “signal pathway*” OR “regulatory mechanism*”).

The molecular and cellular terms in the third search block were intentionally included to focus the bibliometric analysis on the cellular, molecular, and regulatory aspects of echinoderm biomineralization, consistent with the primary scope of this review. No restrictions were applied regarding publication date, language, or document type. The final search retrieved 1300 records.

**Figure 2:**
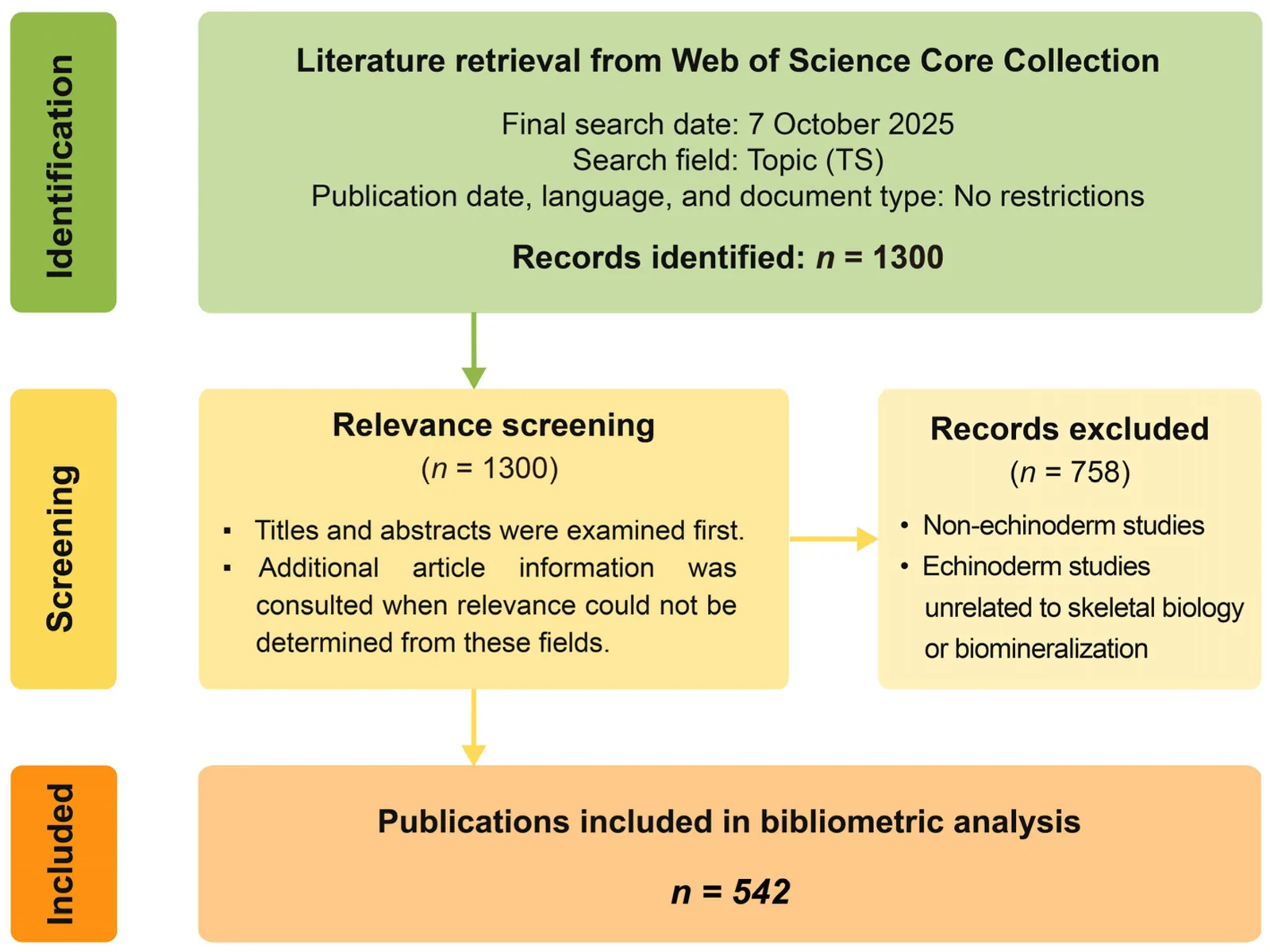
Flow diagram of literature retrieval and screening for the bibliometric analysis. Records retrieved from the Web of Science Core Collection were screened for relevance to the scope of this review, resulting in 542 publications included in the final bibliometric dataset.

The retrieved records were screened by one author for relevance to the scope of this review. Titles and abstracts were examined first, and additional article information was consulted when relevance could not be determined from these fields [37]. Records were included if they focused on echinoderms and addressed biomineralization or skeletal biology, including skeletal formation and calcification, skeletal morphology and structure, skeletogenic cell development, or associated cellular, molecular, and regulatory mechanisms. Studies examining environmental effects were included when they directly assessed skeletal development, calcification, or biomineralization. Studies of non-echinoderm taxa and studies involving echinoderms but without a relevant connection to skeletal biology or biomineralization were excluded. After screening, 758 records were excluded, and 542 publications were retained for bibliometric analysis. The complete list of the included publications is provided in Table S1. The full records and cited references of the included publications were exported in plain-text format for subsequent analysis [38]. The literature retrieval and screening process is summarized in Figure 2.

### 2.2. Bibliometric Analysis

The retrieved bibliographic data were analyzed and visualized using the bibliometrix R package (version 5.0) and CiteSpace (version 6.4.R1) [35,36]. We first used bibliometrix to obtain basic information about the literature data, the distribution of publication and citation volumes over time, and a global output distribution map, and to analyze core source journals, providing essential support for subsequent analysis and research. Subsequently, CiteSpace was used to conduct scientometric analysis on literature from 1985 to 2025. Of the 542 publications included in the complete bibliometric dataset (1937–2025), 528 (97.4%) were published between 1985 and 2025, whereas only 14 publications (2.6%) were published before 1985 and were sparsely distributed across years. Therefore, the complete dataset was used to characterize the overall publication trends, while the 1985–2025 subset was used for CiteSpace analyses.

Across CiteSpace network analyses, the link retaining factor (LRF) was set to 2.5, the maximum links per node (L/N) to 10, the look back years (LBY) to 5, and e to 1.0. Cooperation networks were constructed to examine collaboration patterns among countries and institutions. Country and Institution were selected as the node types. The country collaboration network used a time slice of 1 year and the top 10 items per slice, whereas the institution collaboration network used a time slice of 2 years and the top 5 items per slice. No pruning was applied to the country or institution collaboration networks. Country and institution collaboration networks were generated directly from the affiliation information in the WoS records, with co-occurrence in the same publication representing a collaborative relationship [35]. No additional fractional weighting, manual merging, or standardization of institutional name variants, multicampus entities, or historical affiliations was applied. Node size represents the publication volume, and link thickness indicates the cooperation intensity.

Before keyword analyses, keywords were manually reviewed and standardized using an alias file in CiteSpace. Where appropriate, singular and plural forms, spelling and expression variants, and selected taxonomic or related terms were grouped under representative keywords to reduce terminological redundancy and facilitate thematic interpretation. Keyword was selected as the node type, with a time slice of 1 year and the top 10 items selected per slice. Pathfinder pruning was applied to both individual time slices and the merged network. Keyword co-occurrence network analysis was performed, followed by keyword burst analysis. For burst detection, α_1_/α_0_ and α_i_/α_i−1_ were both set to 2.0, the number of states was set to 2, γ was set to 0.5, and the minimum burst duration was set to 2 years. Keyword clustering was performed, and a keyword timeline view was generated based on the clustering results to identify temporal changes in research topics. Cluster labels were initially generated using the log-likelihood ratio (LLR) method and were subsequently manually refined based on the keywords within each cluster to improve their thematic interpretability. Node size represents the frequency of keyword occurrence, and link thickness indicates the frequency of keyword co-occurrence. Nodes with higher centrality are highlighted with a pink outer ring, where the thickness of the outer ring indicates the strength of betweenness centrality [39]. The remaining statistical data were plotted using Origin 2024 software.

### 2.3. Cross-Genomic Analysis

We selected 14 echinoderm species representing all five extant classes for comparative genomic analysis. We prioritized well-studied representative species for which publicly available genome assemblies and corresponding annotated protein datasets were available. Where multiple candidate species or genomic resources were available, genomes with annotations available in the Reference Sequence (RefSeq) database, assemblies supported by published genome studies, and chromosome-level or otherwise more contiguous assemblies were given priority where possible. We also sought to include representatives from different orders within each class, subject to the availability and quality of annotated genomic resources. Genome accessions and assembly versions, annotation versions or releases, completeness metrics, data sources, retrieval dates, and genome references are provided in Table S2. Among them, *S*. *purpuratus*, *Lytechinus variegatus*, and *Diadema setosum* represent Echinoidea (sea urchins); *Apostichopus japonicus*, *Holothuria leucospilota*, *Holothuria scabra*, and *Chiridota heheva* represent Holothuroidea (sea cucumbers); *Asterias rubens*, *Acanthaster planci*, and *Patiria miniata* represent Asteroidea (starfish); *Amphiura filiformis* and *Ophiura sarsii* represent Ophiuroidea (brittle stars); and *Anneissia japonica* and *Antedon mediterranea* represent Crinoidea (sea lilies/feather stars). At a finer taxonomic scale, broader representation within Echinoidea was also considered. Although cidaroid sea urchins are developmentally informative for comparisons with euechinoids [22,40], the cidaroid genomic resources evaluated in this study did not provide annotated protein datasets comparable to those used in the standardized workflow using the Basic Local Alignment Search Tool (BLAST) for protein–protein searches (BLASTP); therefore, no cidaroid species was included.

To define the genes examined in the comparative analysis, we assembled a targeted candidate set based on Echinobase annotations [41] and published studies of echinoderm skeletogenesis and biomineralization [28,32,42]. The candidate set comprised transcription factors, signaling-related genes, and biomineralization genes and was intended to represent genes with previously reported or putative relevance to skeletal formation and its regulation, rather than a comprehensive inventory of all genes involved in echinoderm biomineralization. Protein sequences from *S*. *purpuratus* were preferentially used as queries because the skeletogenic gene regulatory network and its associated effector genes have been most extensively characterized in this species [12,28]. For genes for which a suitable *S*. *purpuratus* query sequence was not available in the reference set used here, annotated vertebrate protein sequences retrieved from the National Center for Biotechnology Information (NCBI) Gene database were used as queries. The query species, protein accession numbers, and sequence versions for all genes, including the non-echinoderm queries, are provided in Table S3.

For each species, the longest protein isoform of each annotated gene was extracted using TBtools-II v2.475 [43] to construct a protein dataset for BLASTP (BLAST+ v2.12.0) searches. Query proteins were searched against these datasets with an E-value threshold of 1 × 10^−10^. Sequence identity, alignment length, and query and subject coverage were recorded for the retrieved hits. The BLASTP results for the candidate sequences, including repeated hits retrieved by different queries, are provided in Table S4. Candidate sequences were then evaluated using phylogenetic trees generated through the TBtools Blast Zone workflow and conserved-domain annotations from NCBI CD-Search [44] and InterPro [45]. For phylogenetic analysis, selected protein sequences were aligned using MUSCLE v5.1 with the -align option and default settings of two consistency iterations (-consiters 2) and 100 refinement iterations (-refineiters 100). The alignments were trimmed using trimAl v1.4.rev15 with the -automated1 option. Maximum-likelihood trees were inferred using IQ-TREE v1.6.12, with the best-fitting amino acid substitution model selected separately for each alignment using the built-in ModelFinder module (-m MFP); branch support was assessed using 1000 ultrafast bootstrap replicates (-bb 1000) with the -bnni refinement option. No outgroup was specified, and the trees were treated as unrooted. The number and composition of sequences included in each tree were adjusted according to the candidates being examined. BLAST matches, tree placement, and domain composition were considered together when assigning sequences to putative homolog groups. When multiple queries retrieved the same protein within a pooled gene-family category, that protein was counted once. The resulting counts represent putative homologs detected with this workflow. Representative phylogenetic trees are presented in Figures S1–S4.

## 3. Mapping the Research Landscape of Echinoderm Biomineralization

### 3.1. Overview of Literature Characteristics

**Figure 3:**
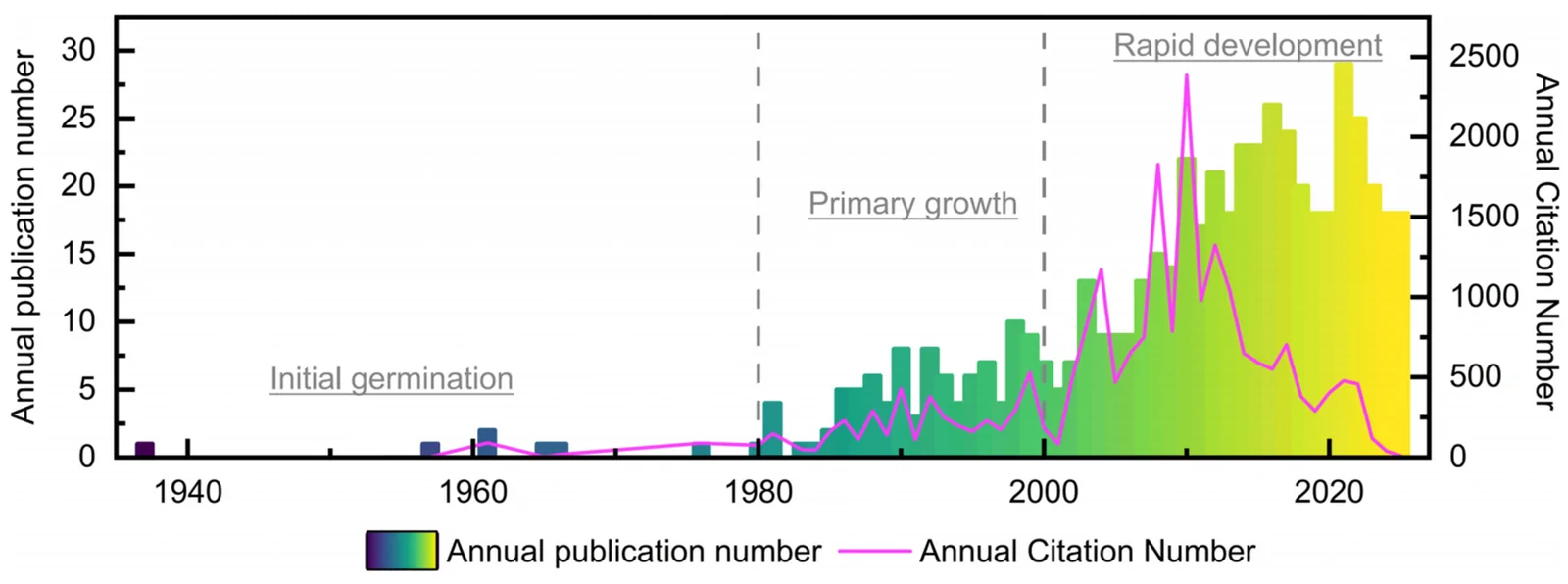
Annual variations in the number of publications and citations. Bars show the number of publications each year. The pink line represents the number of citations each year.

The complete dataset comprised 542 publications on echinoderm biomineralization published between 1937 and 2025 and was analyzed using bibliometrix. The annual publication growth rate was 3.34% over this period, reflecting the historical accumulation and steady development of research in this field. The number of publications directly reflects the development of a research field and acts as a measurable indicator for evaluating the progress of a specific theme [34]. Overall, research in this field started in the late 1930s, remained sporadic for decades, increased gradually after the 1980s, and showed a significant upward trend after 2000 (Figure 3). The annual publication trend falls into three approximate stages: (1) initial stage (1937–1979); (2) growth stage (1980–1999), when publication numbers started to rise; and (3) rapid development stage (2000–2025), during which research output increased sharply and remained high. Citation peaks also emerged during this stage. The lower citation counts for more recent publications may partly reflect citation lag, as these studies have had less time to accumulate citations. In terms of document types, journal articles dominated (482 papers), followed by review articles (32 papers) and a small number of other types, including book chapters and conference papers. This shows that journal articles are the primary medium of research output in this field. The average number of citations per paper was 40.38 (Table S5).

**Figure 4:**
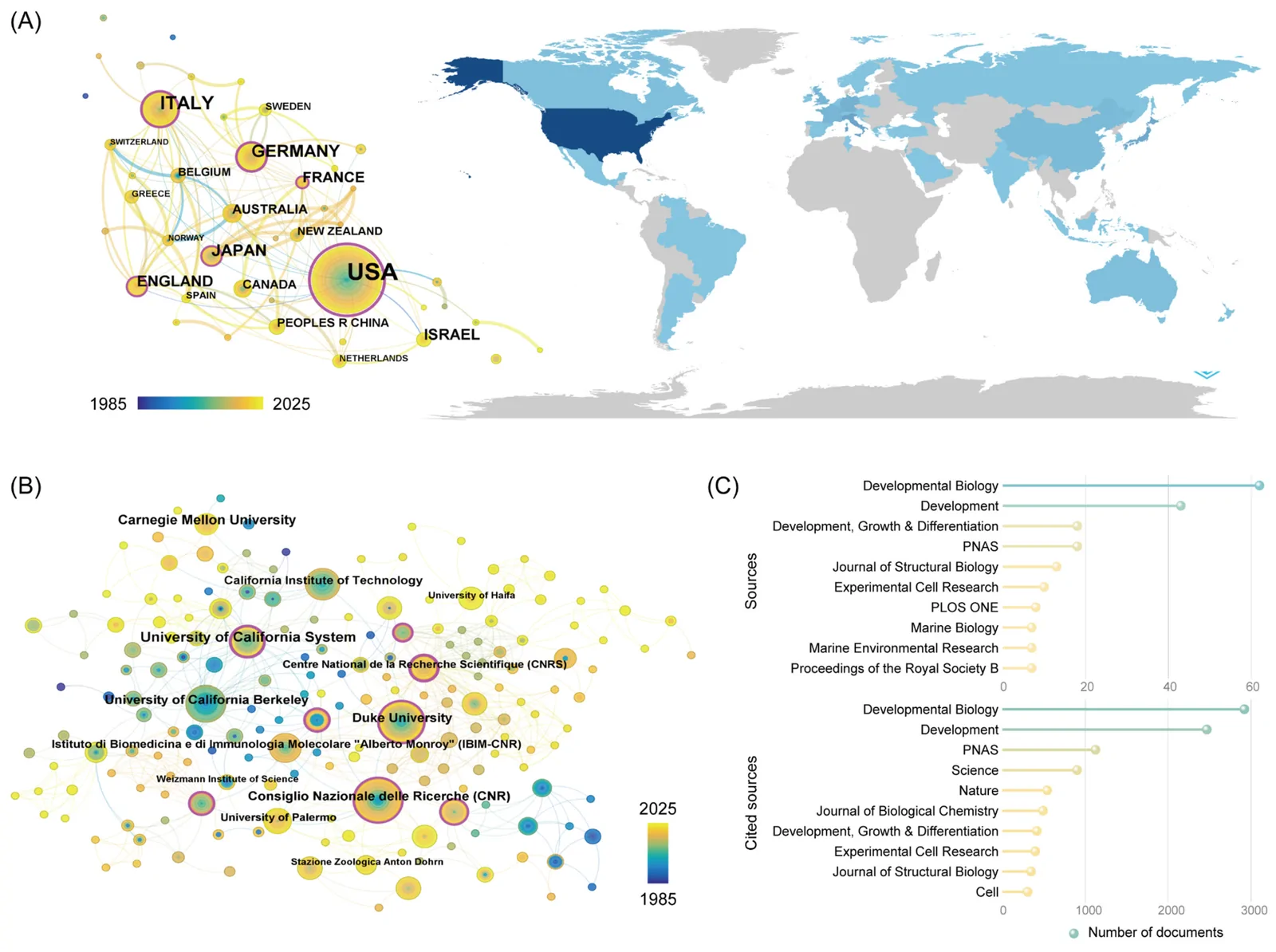
Global distribution of research, institutional collaborations, and journal sources. (**A**) Global national distribution of publications based on the complete dataset (1937–2025, *n* = 542) and international collaboration network generated using CiteSpace (1985–2025, *n* = 528). On the world map, darker blue indicates higher publication output and grey indicates countries or regions for which no publication count was available in the mapped data; (**B**) major research institutions and their collaborative relationships using CiteSpace (1985–2025, *n* = 528); (**C**) publication counts by source journal (top) and counts of cited references by journal (bottom), based on the complete dataset (1937–2025, *n* = 542). Colors reflect the corresponding counts, with yellow indicating lower values and cyan indicating higher values. Node size represents the number of publications, and line thickness indicates collaboration strength. Nodes with higher betweenness centrality are highlighted with pink outer rings, where the thickness of the outer ring denotes the strength of betweenness centrality. Abbreviations: United States of America (USA); Proceedings of the National Academy of Sciences of the United States of America (PNAS).

Figure 4 presents the global distribution and collaboration patterns identified from the bibliometric dataset. The country and institutional collaboration networks generated using CiteSpace were based on the 528 publications published between 1985 and 2025 (Figure 4A,B). The United States of America (USA) led in both publication output and centrality, followed by Italy, Germany, France, and Japan. Countries with high centrality (≥0.1), including the USA, Italy, and Germany, held key positions in the collaboration network, which reflects their core role in knowledge dissemination (Figure 4A). Among research institutions, Duke University, Consiglio Nazionale delle Ricerche (CNR), and the University of California System exhibit high research productivity and frequent international collaboration. However, the overall institutional collaboration density remains low, with no extensive cooperative clusters (Figure 4B). Key publication sources include Developmental Biology, Development, and Journal of Structural Biology. For cited journals, Science, Nature, Proceedings of the National Academy of Sciences of the United States of America, and Developmental Biology were the most frequently cited. This indicates that research in this field relies heavily on highly influential academic studies. Developmental Biology ranks first both in publication volume and citation frequency, highlighting its leading academic role in this field (Figure 4C). Overall, research strength is concentrated in developed European and American countries. High-output institutions here maintain close collaborations, forming a relatively stable international research network.

### 3.2. Research Hotspots and Evolution of Research Frontiers

**Figure 5:**
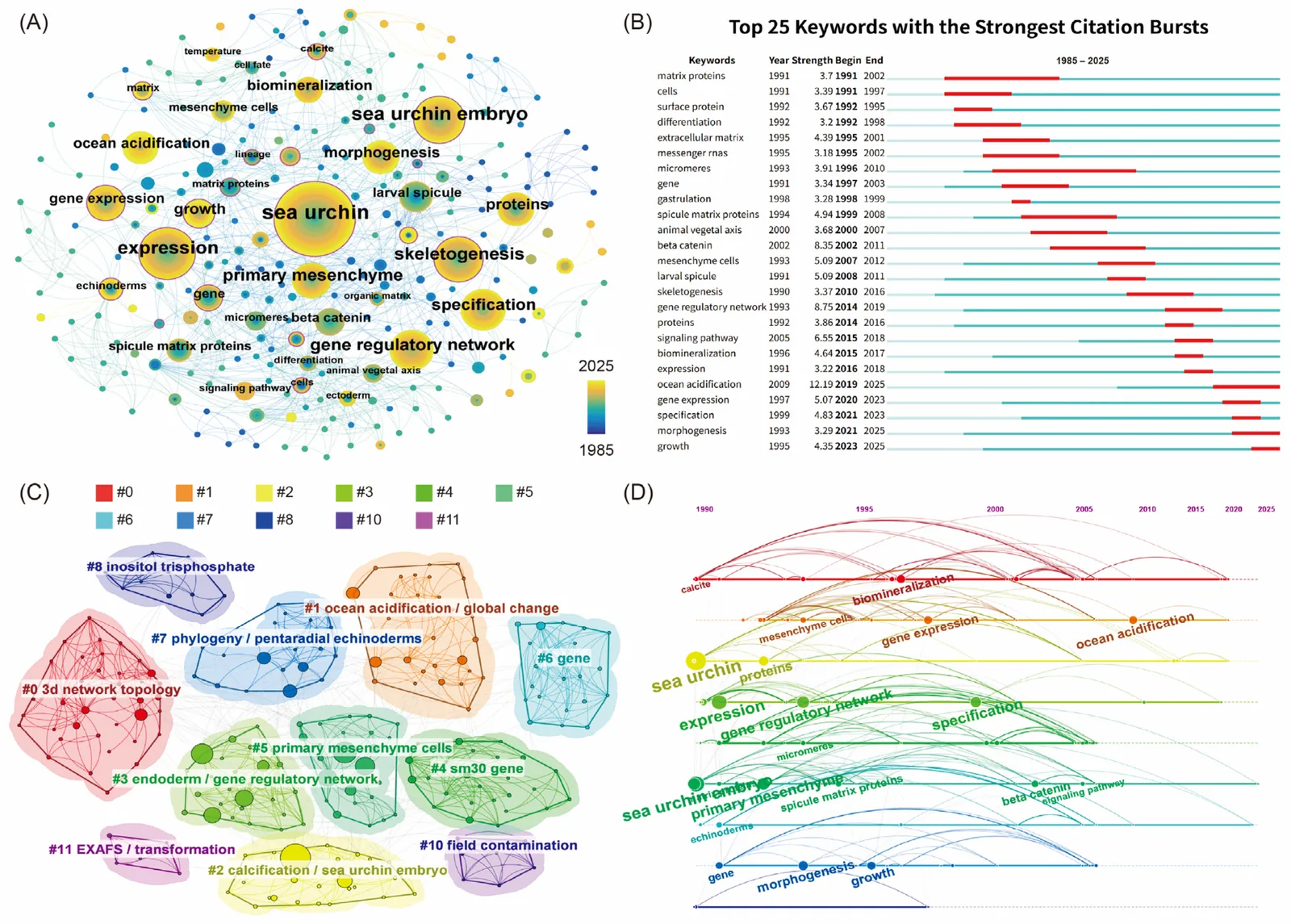
Keyword relevance analysis. (**A**) Keyword co-occurrence network, with node colors ranging from blue (1985) to yellow (2025) representing temporal changes; (**B**) top 25 keywords with the strongest citation bursts, where red bars indicate the duration of the burst; (**C**) keyword clustering (left, different colors represent different cluster categories); and (**D**) keyword temporal trends (right, curves show the temporal distribution of keywords). All CiteSpace analyses shown in this figure were based on publications from 1985 to 2025 (*n* = 528). Node size represents the frequency of keyword occurrence, and line thickness indicates keyword co-occurrence frequency. Nodes with higher betweenness centrality are highlighted with pink outer rings, where the thickness of the outer ring denotes the strength of betweenness centrality.

Keyword-based visual analyses identified major research hotspots and temporal trends within the literature analyzed here (Figure 5). The keyword co-occurrence network (Figure 5A) showed that “sea urchin,” “biomineralization,” “gene regulatory network”, and “primary mesenchyme cells” were the most frequent and central terms. This confirms that sea urchin skeletogenesis and gene regulation mechanisms are long-standing core research topics. Other frequent keywords, such as “expression,” “skeletogenesis,” and “morphogenesis,” correspond to research fields focused on embryonic development and cell differentiation.

Keyword bursts reflect both the intensity and duration of attention to a specific keyword, with longer bursts indicating more sustained research interest (Figure 5B). As shown in Figure 5B, “ocean acidification” showed the highest burst strength (12.19), with a burst spanning 2019–2025, highlighting its prominence in recent research within the analyzed literature. The second strongest burst keyword is “gene regulatory network” (8.75, 2014–2019), which served as a major research frontier during this period. Additionally, “β-catenin” (8.35, 2002–2011) and “mesenchyme cells” (5.09, 2007–2012) also show notable burst strength, identifying them as key research hotspots in the early 2000s. Using the time series of keyword bursts, we can divide the evolution of research hotspots into three stages. In the 1990s, the emerging keywords were mainly “cells,” “surface proteins,” and “differentiation,” focusing on the basic cell biology of echinoderms. From the early 21st century to 2015, the emerging keywords turned to “β-catenin”, “ossicle matrix proteins”, and “larval ossicles”, focusing on the molecular mechanisms of skeletal development in echinoderms. Since 2015, the emerging keywords have included “gene regulatory networks”, “signaling pathways”, and “ocean acidification”. They focus on the molecular regulatory networks of biomineralization and the responses to global environmental changes.

Keyword clustering analysis (Figure 5C) identified 11 major clusters, which cover multiple levels from molecular regulation to environmental response. The clustering yielded a modularity Q of 0.6858 and a weighted mean silhouette S of 0.894. The sizes of the 11 major clusters ranged from 5 to 38 keyword nodes, with individual silhouette values ranging from 0.838 to 0.995. Cluster #0 focuses on the structural features of echinoderm skeletons, particularly sea urchin spines. Cluster #2 (“calcification/sea urchin embryo”) focuses on the calcification process in sea urchin embryonic development. Among them, Cluster #4 (*sm30* gene) and Cluster #5 (primary mesenchyme cells) are directly linked to biomineralization genes and osteogenic cell differentiation. They highlight the role of primary mesenchyme cells in echinoderm skeletogenesis. Clusters #3 (“gene regulatory network”) and #6 (“gene”) focus on the regulatory functions of gene networks in echinoderm biomineralization and development. Thus, genes, regulatory networks and cellular differentiation represent prominent themes within the literature analyzed here. Cluster #1 (“Ocean acidification/global change”) focuses on the impact of global environmental changes (especially ocean acidification) on echinoderm biomineralization. It reflects the influence of environmental changes on the mineralization process. Since 2019, attention to this topic has increased, establishing it as a current research frontier in the field. Overall, research themes have gradually expanded from pure developmental biology to the intersection of ecology and molecular biology.

The timeline view (Figure 5D) shows continuous and overlapping relationships among themes. “Biomineralization” and “gene regulatory network” remain consistently active, while the emergence of “ocean acidification” indicates a gradual shift from embryonic development to ecological and environmental regulation. Overall, sea urchin biomineralization research presents an evolutionary path: from structural description to genetic mechanisms, and then to environmental responses. This reflects interdisciplinary integration and dynamic progress of research frontiers.

The search strategy was intentionally designed to emphasize the cellular, molecular, and regulatory aspects of echinoderm biomineralization, consistent with the focus of this review. Therefore, the bibliometric results primarily characterize research within this defined scope. Nevertheless, because the bibliometric dataset was derived exclusively from the Web of Science Core Collection, relevant studies indexed only in other databases may not have been captured. In addition, raw citation counts were not normalized by publication year, and the incomplete coverage of 2025 should be considered when interpreting citation patterns and the most recent research fronts.

## 4. Cellular and Gene Regulatory Basis of Echinoderm Skeletogenesis

Our bibliometric analysis identified skeletogenic cell differentiation, primary mesenchyme cells (PMCs), and gene regulatory networks (GRNs) as major and persistent themes within the literature analyzed here (Figure 5). The prominence of these topics largely reflects the importance of sea urchin embryos as experimental models for studying cell specification, morphogenesis, and biomineral formation. In particular, the euechinoid PMC lineage provides one of the most extensively characterized developmental GRNs in any animal embryo. However, the available evidence is taxonomically uneven. Causal regulatory interactions have been studied mainly in a few sea urchin species, whereas research in other echinoderm classes is often based on gene expression, comparative transcriptomics, or perturbation of selected regulatory components. Therefore, the sea urchin PMC system provides an essential mechanistic framework, but it should not be treated as a GRN that operates unchanged across all echinoderms.

### 4.1. Developmental Diversity of Echinoderm Skeletogenic Mesenchyme Cells

In euechinoid sea urchins, larval skeletogenesis initiates with the specialization and migration of primary mesenchyme cells (PMCs) [46]. As a key cell type in skeletal development, PMC formation provides the basis for subsequent biomineralization and skeletal patterning [29]. During the fourth embryonic cleavage, an unequal division at the vegetal pole produces four micromeres. At the fifth cleavage, these micromeres divide asymmetrically to generate four large micromeres and four small micromeres. The large micromeres subsequently give rise to the PMC lineage [47,48]. These large micromeres subsequently specify as the skeletal lineage in the vegetal pole region [49]. PMC fate depends mainly on the autonomy of maternal signals [49]. Nuclear β-catenin provides an important early input into the vegetal regulatory state, while transcription factors such as Otx participate in the activation of the downstream biomineralization genes [12,28].

At the blastula stage, PMCs undergo epithelial–mesenchymal transition (EMT). They detach from the epithelial layer, migrate into the blastocoel, and move along the inner wall, eventually forming the ventrolateral ring structure [13]. During migration, PMCs extend interconnected filopodia to form a syncytium-like 3D cellular network [50]. Skeletal precursor deposition initiates in the interstices of this network [51]. Ectodermal signals provide important spatial information during these processes. Vascular endothelial growth factor (VEGF) and fibroblast growth factor (FGF) signaling regulate PMC migration, positioning, and skeletal morphogenesis, whereas Wnt5 contributes to the establishment of ectodermal patterning cues that influence posterior skeletal development [52,53]. PMCs express the corresponding receptors, including vascular endothelial growth factor receptor (VEGFR) and fibroblast growth factor receptor (FGFR), allowing ectodermal patterning information to be translated into localized cellular behavior and skeletal growth [54,55,56].

However, the developmental origin and behavior of embryonic skeletogenic cells are not uniform across echinoderms. In euechinoids, the larval skeleton is secreted by the primary mesenchyme cells (PMCs), a lineage that arises early from micromeres and is described above [57]. Cidaroids constitute one of the two major extant echinoid lineages and are the sister lineage to euechinoids [40]. Their phylogenetic position and distinct developmental features make them particularly informative for comparative studies of the evolution of echinoid skeletogenesis [22,40]. The skeletogenic mesenchyme cells of cidaroids do not ingress prior to archenteron invagination; instead, both skeletogenic and non-skeletogenic mesenchyme cells initiate migration into the blastocoel concomitantly after the onset of gastrulation [22]. In holothuroids, skeletogenic mesenchyme cells arise from the vegetal plate and ingress prior to gastrulation, forming a relatively simple embryonic skeletal structure [12]. Ophiuroids exhibit an even earlier and more extensive ingression of skeletogenic cells before gastrulation, producing a larger population of mesenchymal cells that contribute to a more elaborate larval skeleton [58,59]. By contrast, asteroids lack an early skeletogenic mesenchyme lineage and do not form a larval skeleton [12]. Research on crinoids is limited, but it is clear that they lack larval skeletons [12]. These comparisons indicate that similar skeletogenic cell functions can develop from different embryonic lineages and through different developmental routes. Therefore, “skeletogenic mesenchyme cells” is the more appropriate term for cross-class comparisons, whereas “PMC” should be used mainly for the specialized micromere-derived skeletogenic lineage of euechinoids.

### 4.2. Hierarchical Structure and Biological Significance of the Gene Regulatory Network (GRN)

The specification and differentiation of PMCs are controlled by a hierarchical GRN that converts maternal polarity information into a skeletogenic transcriptional program. During skeletogenesis, the skeletal GRN regulates cell fate, gene activation, and spatial pattern formation. It acts as a core framework for understanding the biomineralization mechanisms in echinoderms [60,61]. This network translates maternal signals and axial information into gene expression programs specific to certain cell types through multilayer transcriptional regulation, thus achieving precise control over skeletal formation [12].

**Figure 6:**
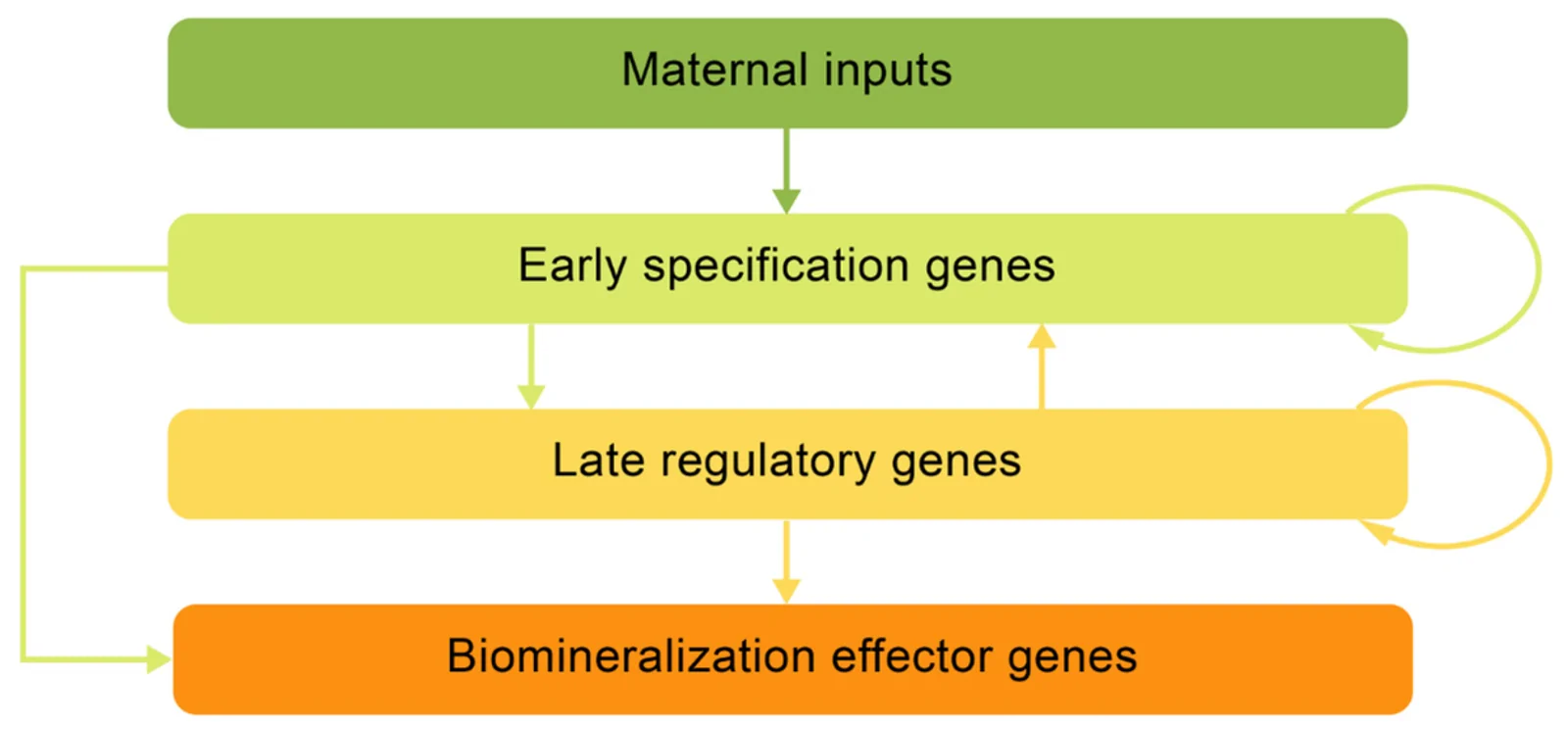
Conceptual hierarchy of euechinoid skeletogenic regulation. Boxes show regulatory levels; arrows indicate schematic flow and feedback, not verified gene-to-gene interactions [12,28].

In early euechinoid development, maternal factors β-catenin and Otx accumulate in the vegetal pole. They define micromere developmental potential and initiate the downstream *pmar1*/*hesC* double-negative gate (DNG) [62]. Within this module, Pmar1 represses transcription of *hesC*. This relieves HesC-mediated suppression of skeletal lineage genes, triggering the skeletal fate program [63]. This mechanism ensures the directionality and irreversibility of lineage specialization, rendering it the earliest activated key node in the GRN. However, it represents a well-resolved feature of the euechinoid GRN and should not be presented as a universal echinoderm mechanism. Upon DNG activation, the GRN proceeds to the lineage specialization stage. A set of core transcription factors are activated sequentially, with Alx1 acting as the central regulator of PMC fate [64]. Expression of *alx1* is regulated by the DNG module and is maintained through positive feedback. The downstream targets of Alx1, together with Ets1, regulate numerous skeletal effector genes [65]. The feedforward loop formed between these factors enhances gene expression stability, ensuring precise execution of the skeletogenesis program in a complex signaling environment [12]. As the hierarchical GRN cascades downward, genes encoding skeletal structural matrix proteins (*pm27*, *sm29*, *sm32*, *sm37*, and *sm50*) and cell behavior-related genes are activated sequentially [66,67]. These genes integrate cellular functions, including mineralization, adhesion, and migration [68]. Ectodermal signaling molecules (VEGF, FGF, and Wnt5) regulate PMC migration direction and skeletal patterning via their respective receptor pathways. This further strengthens the coupling of morphological development and molecular regulation [53,54,69]. The biomineralization-related branch of this hierarchical regulatory organization in euechinoids is summarized schematically in Figure 6.

The skeletal GRN exhibits three key systematic features: robustness, modularity, and plasticity [30]. Multilayered feedback and feedforward structures bolster network stability, while its modular organization enables reuse of functional units across diverse developmental contexts [31,70]. Some core modules can be reactivated during adult skeletal regeneration and metamorphosis, reflecting the GRN’s high evolutionary plasticity [29]. Furthermore, if PMCs are removed or key genes (e.g., *alx1*) are inhibited, other cells can still reactivate specific GRN modules and regain mineralization capacity [13]. This “network rewiring” phenomenon indicates that cell fate is determined not only by genetic makeup but also by the dynamic state of the regulatory network [71,72].

Comparative developmental studies suggest that echinoderm skeletogenesis involves both shared regulatory components and lineage-associated modifications. Regulatory factors such as Alx1 and Ets1, together with signaling components such as VEGFR and FGFR, participate in skeletogenic development in multiple echinoderm groups [12,15,73,74]. Alx1 is particularly important because its association with skeletogenic cells extends beyond the euechinoid PMC lineage. Functional and expression studies in sea cucumbers, brittle stars, and sea stars support an ancestral role for Alx-family transcription factors in echinoderm skeletogenesis, followed by their recruitment into the embryonic PMC program of sea urchins [75].

At the same time, several features of the euechinoid GRN are not broadly conserved. The early segregation of a micromere-derived PMC lineage is specific to sea urchins, and the *pmar1/hesC* double-negative gate has not been demonstrated as a universal mechanism outside euechinoids [12]. Even within echinoids, changes in the regulation of *alx1* and other early specification genes indicate substantial rewiring between euechinoid and cidaroid lineages [71]. Other regulatory genes can also be deployed in different developmental territories. For example, *tbr* functions in the skeletogenic micromere lineage of sea urchin embryos, whereas in sea star embryos it operates in the vegetal endomesoderm and is required for archenteron formation [76]. Thus, the presence of orthologous transcription factors does not necessarily imply conservation of their developmental inputs, downstream targets, or network positions. Several transcription factor genes, including *ets1*, *tbr*, and *hesC*, as well as differentiation genes associated with sea urchin skeletogenesis, are also expressed during brittle star skeletogenesis, although their expression timing and inferred regulatory relationships differ [59]. These findings support the reuse of an ancestral skeletogenic toolkit while also indicating that the developmental inputs and regulatory connections controlling this toolkit have changed among lineages.

Taken together, current evidence supports a model of partial regulatory conservation rather than a single GRN that is fully conserved across the phylum. Some transcription factors, signaling pathways, and differentiation genes are repeatedly associated with skeletogenesis, but their network connections, expression timing, and cellular contexts can differ. This distinction is important because most experimentally resolved regulatory interactions still come from euechinoid sea urchins. The prominence of PMC and GRN research in the bibliometric results, therefore, reflects both a major mechanistic achievement and a strong taxonomic concentration of existing knowledge. These functional studies provide the biological framework for interpreting the cross-class distribution and lineage-associated variation of biomineralization genes presented in the following section.

## 5. Broadly Distributed Skeletogenic Regulatory Components and Variation in Biomineralization Genes Across Echinoderms

**Figure 7:**
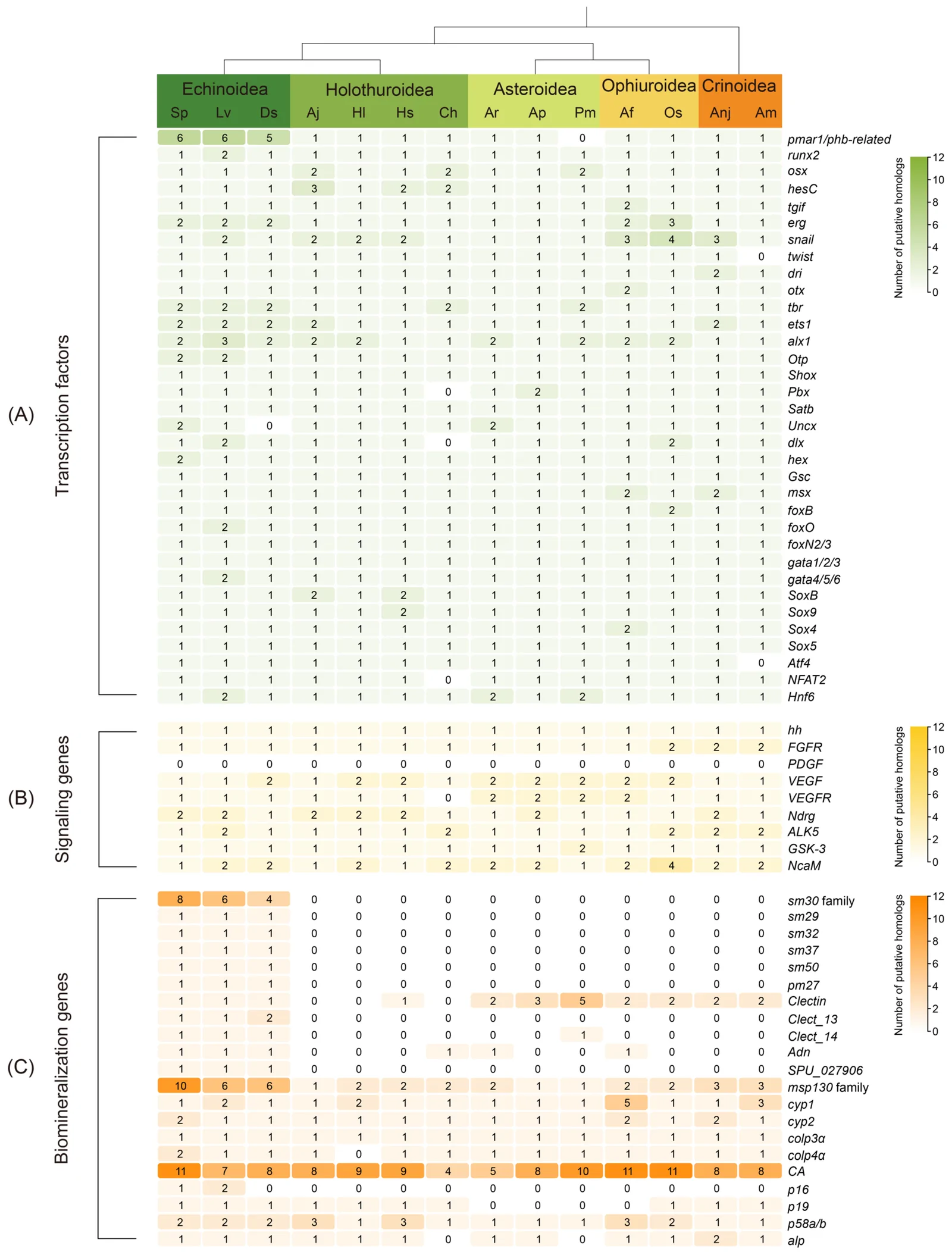
Heatmap showing the distribution of (**A**) transcription factor genes, (**B**) signaling-related genes, and (**C**) biomineralization genes across the examined echinoderm species. Values indicate the numbers of distinct putative homologs identified for each gene or gene group. A value of 0 indicates that no putative homolog was detected under the present search criteria. The *sm30* family row combines *sm30A–F*, the *msp130* family row combines the six *msp130* query sequences, and the *p58a/b* row combines *p58a* and *p58b*. The species phylogeny follows [18]. Sp, *Strongylocentrotus purpuratus*; Lv, *Lytechinus variegatus*; Ds, *Diadema setosum*; Aj, *Apostichopus japonicus*; Hl, *Holothuria leucospilota*; Hs, *Holothuria scabra*; Ch, *Chiridota heheva*; Ar, *Asterias rubens*; Ap, *Acanthaster planci*; Pm, *Patiria miniata*; Af, *Amphiura filiformis*; Os, *Ophiura sarsii*; Anj, *Anneissia japonica*; Am, *Antedon mediterranea*.

Our cross-class comparative analysis revealed contrasting distribution patterns among regulatory components and biomineralization genes (Figure 7). Most transcription factor genes associated with skeletogenesis, including *otx*, *SoxB*, *alx1*, *tbr*, and *ets1*, were identified across the major echinoderm classes (Figure 7A), and many signaling-related genes showed similarly broad distributions (Figure 7B). These patterns indicate that many regulatory components implicated in the well-characterized skeletogenic GRN of euechinoid sea urchins are broadly distributed across echinoderms. However, the presence of homologous regulatory genes does not demonstrate conservation of their expression, function, regulatory interactions, or network topology. Comparative developmental studies have shown substantial changes in the deployment and connectivity of skeletogenic regulatory genes among echinoderm lineages [58,59,71]. Thus, our comparative genomic results support the presence of a broadly shared regulatory toolkit rather than a fully conserved biomineralization GRN.

Biomineralization genes showed substantially greater variation among classes than the regulatory components (Figure 7C). Several biomineralization genes, including carbonic anhydrase genes (*CA*), cytochrome P450 genes (*cyp*), collagen-related genes (*colp3/4α*), and *p58*, were identified across the examined echinoderm classes, suggesting that some components associated with biomineralization are widely distributed. In contrast, several spicule matrix genes, including *sm29*, *sm32*, *sm37*, and *sm50*, were predominantly identified in the examined echinoids. This pattern indicates that echinoids contain a broader range of the spicule matrix genes included in our analysis.

Among individual gene families, *msp130* showed one of the clearest differences in the number of identified putative homologs, with consistently higher numbers in the examined echinoids than in representatives of the other four classes. Previous phylogenetic studies have also reported lineage-specific duplication and diversification of the *msp130* family in echinoderms [59]. Together, these observations are consistent with lineage-associated expansion of the *msp130* family in echinoids. Transcription factor families can also undergo lineage-specific diversification. Previous phylogenetic and functional studies distinguish echinoid *pmar1* from non-echinoid *phb* and reveal both gene duplication and changes in the regulatory interactions involving members of the *pmar1/phb*-related family [77]. This provides an example of how broadly distributed regulatory components can undergo family-specific diversification and regulatory rewiring during echinoderm evolution.

The broader taxonomic comparison also provides a different perspective on previously proposed changes in biomineralization gene content. A previous genomic study proposed that the reduced biomineralization of sea cucumbers may be associated with contraction of biomineralization genes [32]. In our cross-class comparative analysis, however, holothuroids, asteroids, ophiuroids, and crinoids showed broadly similar distributions of the biomineralization genes included in the analysis, whereas echinoids were distinguished by the presence of several additional spicule matrix genes. This broader pattern suggests that the differences between echinoids and holothuroids may reflect lineage-associated diversification of biomineralization genes in echinoids, rather than contraction in holothuroids alone. These comparative patterns should be interpreted within the scope of the present analysis. Although the genome assemblies used here were generally of high completeness (Table S2), most query sequences were from *S*. *purpuratus*, and sequence divergence among echinoderm lineages and differences in genome annotation may have affected the detection of highly divergent or incompletely annotated homologs. Accordingly, apparent absences and differences in the number of detected putative homologs were treated as descriptive patterns rather than definitive evidence of gene loss or statistically supported gene-family expansion or contraction.

Overall, our comparative analysis indicates that echinoderm biomineralization is characterized by broadly distributed regulatory components together with greater lineage-associated variation in biomineralization genes. While many transcription factors and signaling-related genes are distributed across the major echinoderm classes, several biomineralization genes and gene families show marked differences in distribution and, in some cases, in the number of identified homologs. These patterns support a model in which a broadly shared regulatory toolkit coexists with lineage-associated diversification of biomineralization genes and individual transcription factor families [59,71,77]. However, the regulatory interactions among these components have been experimentally resolved mainly in euechinoid sea urchins [12,59,71], and further functional studies in other echinoderm classes are needed to determine how these genomic differences relate to skeletal development.

## 6. Future Research Considerations

Future research should address several connected gaps in echinoderm biomineralization, including taxonomic sampling, identification of cell types, causal regulatory mechanisms, differences among life stages, and environmental responses. Addressing these gaps is necessary to distinguish broadly shared mechanisms from those restricted to particular lineages, developmental stages, or environmental conditions. We therefore highlight the following five research priorities:

1. Broadening taxonomic and genomic coverage of echinoderm biomineralization research. Current functional and mechanistic evidence remains heavily concentrated in euechinoid sea urchins, limiting our understanding of biomineralization in holothuroids, asteroids, ophiuroids, and crinoids [12]. Declining sequencing costs, advances in long-read sequencing, and the continued expansion of echinoderm genomic resources now make broader taxonomic sampling increasingly feasible [78,79]. Broader taxonomic sampling is essential for distinguishing conserved biomineralization components from lineage-specific gene changes and for testing whether euechinoid mechanisms apply across echinoderms. Cidaroid sea urchins are particularly important in this regard because their phylogenetic position as the sister lineage to euechinoids makes them highly informative for testing the conservation and modification of skeletogenic mechanisms within Echinoidea [22,40]. However, well-annotated genomic resources suitable for standardized comparative analyses remain limited. Future studies should prioritize high-quality genomes and developmental transcriptomes from diverse species, with improved annotation to distinguish true gene absence or copy number variation from assembly and annotation gaps.
2. Resolving skeletogenic cell identities and developmental origins. Skeletogenic cells in different echinoderm classes do not necessarily originate from micromeres as euechinoid PMCs do, making direct comparisons of their cellular identities and regulatory programs uncertain [15,57,59]. Determining whether these cells share a common developmental origin or have been independently recruited is therefore essential before comparing their GRNs across lineages. Single-cell transcriptomic analyses can identify skeletogenic cell states and infer differentiation trajectories [80,81], whereas spatial transcriptomics and experimental lineage tracing are needed to resolve their tissue locations and developmental origins. Integrating these approaches would provide the cellular framework required to distinguish broadly shared skeletogenic cell types from lineage-specific developmental innovations.
3. Experimental validation of gene functions and regulatory connections. The presence or expression of homologous genes does not demonstrate that they retain the same functions or regulatory connections across echinoderm lineages [59,71]. This limitation is important because homologous genes may occupy different positions in developmental GRNs or regulate different downstream targets, meaning that expression comparisons alone may overestimate regulatory conservation [48,71]. Functional perturbation through gene knockdown, knockout, and ectopic expression is therefore needed to test whether candidate genes retain comparable skeletogenic functions across species [15,26,82]. Cis-regulatory analysis and genome-wide target identification should further be used to distinguish direct regulatory connections from indirect effects and to determine which interactions are conserved, redeployed, or rewired [48,83]. Together, these experiments would provide the causal evidence required to explain how changes in GRN architecture contribute to differences in skeletogenic development among echinoderm lineages.
4. Comparing biomineralization across life stages and skeletal structures. Current understanding of echinoderm skeletogenesis is based mainly on the sea urchin larval skeleton, whereas the formation of juvenile and adult skeletal structures remains less well understood [13,84]. In sea urchins, larval and adult skeletogenic cells have different developmental origins but express several common regulatory and differentiation genes, raising the question of whether they use the same GRN or only share selected components [84,85]. Future studies should combine cell lineage tracing, transcriptomic analysis, and functional perturbation to compare larval, juvenile, and adult skeletogenesis and identify mechanisms shared across life stages and those specific to particular skeletal structures. Such comparisons could test the hypothesis that the larval skeletogenic program arose through the redeployment and modification of an ancestral adult GRN and clarify how biomineralization mechanisms change during the echinoderm life cycle [12,85].
5. Extending comparative biomineralization research to environmental responses. Beyond genetic and developmental mechanisms, our bibliometric analysis identified ocean acidification as a distinct keyword cluster and a prominent theme in recent years, indicating growing research interest in the environmental regulation of echinoderm biomineralization. This bibliometric pattern provides a clear basis for extending the comparative framework to ocean acidification. Ocean acidification is particularly relevant to biomineralization because the absorption of anthropogenic carbon dioxide by seawater lowers pH, carbonate ion concentration, and calcium carbonate saturation state, thereby altering carbonate availability and the physiological conditions required for calcification [86,87]. Experimental studies in echinoderms show that ocean acidification can alter larval growth and skeletogenesis, acid–base regulation, and the expression of biomineralization-related genes [88,89,90]. However, responses differ among species and life stages and depend on the trait measured and the severity and duration of exposure; some traits show no significant change under projected near-future acidification scenarios, whereas adverse effects may become more apparent under stronger or longer exposure [91,92,93]. Despite the increasing research attention revealed by the bibliometric results, experimental evidence remains concentrated in heavily calcified echinoids, whereas less heavily calcified groups, particularly holothuroids, remain poorly represented [90,94]. Future studies should compare several echinoderm species with different levels of calcification under comparable ocean acidification conditions. Measuring acid–base regulation, energy use, biomineralization-related gene expression, and skeletal formation in the same experiments would determine whether acidification sensitivity is mainly associated with calcification level or with physiological differences among lineages [95]. This comparison is important for explaining why some echinoderms show reduced skeletal growth under acidification, whereas others maintain biomineralization [93,96].

## 7. Conclusions 

This review integrates bibliometric analysis, mechanistic evidence, and cross-class genomic comparisons to provide an overview of echinoderm biomineralization research. The field has expanded from morphological descriptions to studies of skeletogenic cells, gene regulatory networks, biomineralization genes, and environmental responses, with primary mesenchyme cells (PMCs), skeletogenic GRNs, and ocean acidification emerging as dominant themes. However, current research remains heavily biased toward sea ur-chins, especially a limited number of euechinoid species, resulting in a detailed but tax-onomically narrow model of skeletogenesis.

Comparative evidence from PMC developmental programs and 14 echinoderm genomes indicates both conservation and diversification in biomineralization mecha-nisms. While many core regulatory components identified in the euechinoid skele-togenic GRN and biomineralization genes are broadly distributed across echinoderms, their deployment and regulatory interactions can differ among lineages, and selected biomineralization gene families show lineage-associated diversification. Overall, echi-noderm biomineralization reflects a combination of shared regulatory elements, line-age-specific genomic evolution, and environmental modulation, rather than a single conserved developmental program.

## Data Availability

The data analyzed in this study were obtained from publicly available databases, as described in Section 2. The data generated or compiled during this study are provided within the article and its Supplementary Materials.

## Supplementary Materials

The following supporting information can be downloaded at https://www.mdpi.com/article/10.3390/biology15201796/s1. Table S1: Publications included in the bibliometric analysis; Table S2: Genomic resources of echinoderm species used in the comparative genomic analysis; Table S3: Query sequences used for homolog identification of biomineralization-related genes in echinoderms; Table S4: BLASTP results supporting the identification of putative biomineralization-related homologs in 14 echinoderm species; Table S5: Main characteristics of the bibliometric dataset used in this study. Figure S1: Protein-sequence phylogenies used to evaluate SM-related candidate genes; Figure S2: Maximum-likelihood phylogenies of MSP130 and related candidate protein sequences; Figure S3: Maximum-likelihood phylogenies of Clectin-related candidate protein sequences; Figure S4: Protein-sequence phylogenies used to evaluate *pmar1*-related candidate genes. References [97,98,99,100,101,102,103,104,105] are cited in Table S2, and references [28,32,42] are cited in Table S3.
