# Supplementary material for: Exploring Biomineralization in Echinoderms: A Review of Gene Regulatory Networks Through Bibliometrics and Comparative Genomics
Source: Biology (Basel). 2026 Oct 9;15(20):1796. doi: 10.3390/biology15201796 (PMC13701902; doi:10.3390/biology15201796)
Supplement: Supplementary file 1 [file biology-15-01796-s001.zip › Supplementary Figures.pdf]

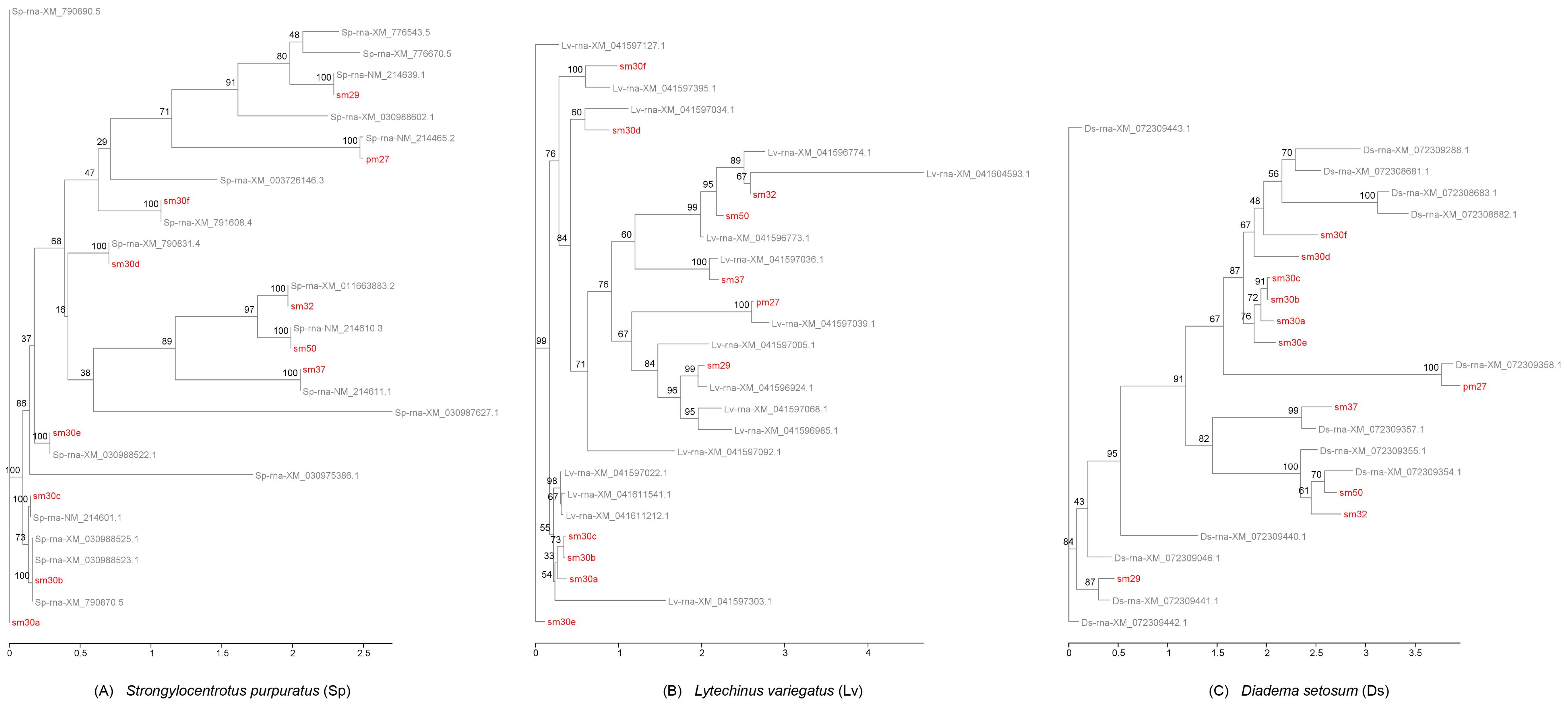


Figure S1. Protein-sequence phylogenies used to evaluate SM-related candidate genes. Separate maximum-likelihood trees were reconstructed for (A) *Strongylocentrotus purpuratus*, (B) *Lytechinus variegatus*, and (C) *Diadema setosum*. Reference protein sequences, including SM and PM27 sequences, are labeled in red; sequences examined from each species are labeled in gray. Numbers at internal nodes indicate ultrafast bootstrap support (%).

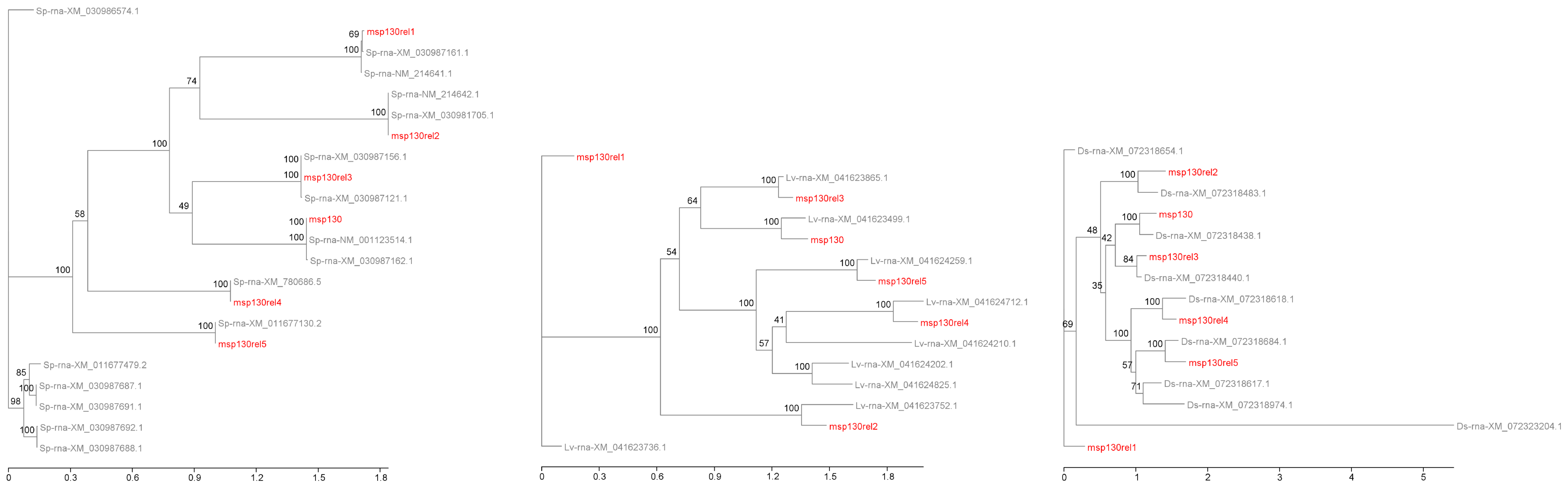


(A) *Strongylocentrotus purpuratus* (Sp) (B) *Lytechinus variegatus* (Lv) (C) *Diadema setosum* (Ds)

Figure S2. Maximum-likelihood trees were inferred separately for (A) *Strongylocentrotus purpuratus*, (B) *Lytechinus variegatus*, and (C) *Diadema setosum*. MSP130 and related reference protein sequences are labeled in red, and sequences examined from each species are labeled in gray. Numbers at internal nodes indicate ultrafast bootstrap support (%).

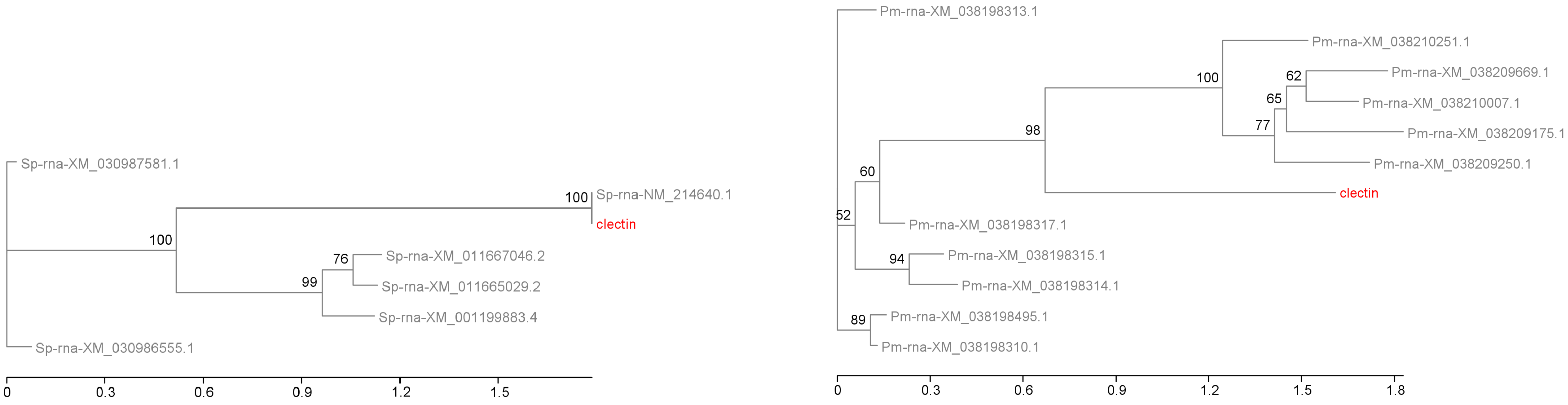


(A) *Strongylocentrotus purpuratus* (Sp)

(B) *Patiria miniata* (Pm)

Figure S3. Maximum-likelihood trees were inferred separately for (A) *Strongylocentrotus purpuratus* and (B) *Patiria miniata*. The Clectin reference protein sequence is labeled in red, and sequences examined from each species are labeled in gray. Numbers at internal nodes indicate ultrafast bootstrap support (%).

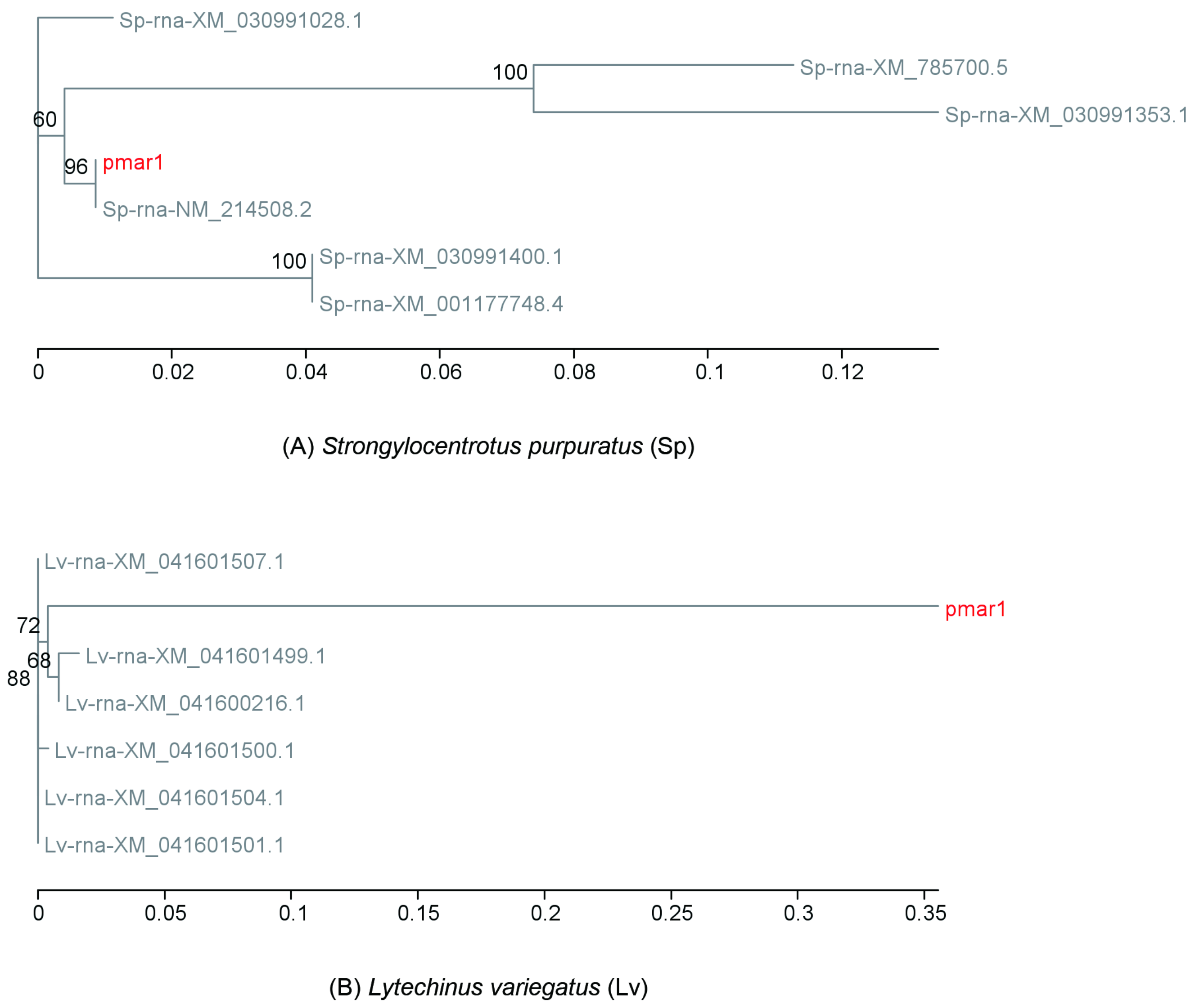


Figure S4. Maximum-likelihood trees were inferred separately for (A) *Strongylocentrotus purpuratus* and (B) *Lytechinus variegatus*. The reference sequence labeled pmar1 is shown in red, and sequences examined from each species are labeled in gray. Numbers at internal nodes indicate ultrafast bootstrap support (%).
